# The Expression of miR-192 and Its Significance in Diabetic Nephropathy Patients with Different Urine Albumin Creatinine Ratio

**DOI:** 10.1155/2016/6789402

**Published:** 2016-01-05

**Authors:** Xiaoyu Ma, Canlu Lu, Chuan Lv, Can Wu, Qiuyue Wang

**Affiliations:** ^1^Geriatrics Department, The First Hospital of China Medical University, Shenyang, Liaoning, China; ^2^Endocrine Department, The First Hospital of China Medical University, Shenyang, Liaoning, China; ^3^Endocrine Department, The People Hospital of Liaoning Province, Shenyang, Liaoning, China

## Abstract

*Objective*. To investigate the expression of miR-192 and its significance in diabetic nephropathy (DN) patients.* Methods*. 464 patients with type 2 diabetes mellitus (T2DM) were divided into normal albuminuria group (NA, *n* = 157), microalbuminuria group (MA, *n* = 159), and large amount of albuminuria group (LA, *n* = 148). 127 healthy persons were selected as the control group (NC, *n* = 127). The serum miR-192 levels were detected by Real-Time PCR and transforming growth factor-*β*1 (TGF-*β*1) and fibronectin (FN) were detected by enzyme-linked immunosorbent assay. The relationships among these parameters were analyzed by Pearson correlation analysis and multiple linear regression analysis.* Results*. The miR-192 in the LA group was significantly lower than other groups, which was lower in the MA group than in the NA group (*P* < 0.01). The TGF-*β*1 and FN in the LA group were significantly higher than other groups, which were higher in the MA group than in the NA group (*P* < 0.01). The expression of miR-192 was negatively correlated with TGF-*β*1, FN, and Ln (UACR) and miR-192, TGF-*β*1, and FN were independent relevant factors affecting Ln (UACR) in T2DM (*P* < 0.01).* Conclusions*. These findings indicate that the levels of miR-192 were lower accompanied by the decrease of urine albumin creatinine ratio (UACR) and the association between miR-192 and nephritic fibrosis in DN.

## 1. Introduction

DN is one of the chronic complications of diabetes and it is the main cause of end-stage renal disease (ESRD). DN is characterized by glomerular basement membrane (GBM) and tubular basement membrane (TBM) thickening, extracellular matrix (ECM) of glomerular mesangial area accumulation, and renal tubule interstitial fibrosis, which cause glomerular sclerosis, proteinuria, and renal failure eventually.

TGF-*β*1 belongs to transforming growth factor superfamily and it can promote renal fibrosis, increase the number of glomerular mesangial cells, and stimulate ECM accumulation in experimental and human DN [1–3]. ECM accumulation can induce further glomerular sclerosis and tubular endothelium fibrosis and cause glomerular filtration rate to progressively decrease and renal failure in DN. Miller et al. [4] found that the TGF-*β*1 mRNA was higher in the glomerulus of DN patients and it is parallel with the glomerular sclerosis extents. Therefore TGF-*β*1 is supposed to be a marker of glomerular sclerosis. FN is a noncollagenous glycoprotein which is the main component of ECM. The ECM consists of collagens, laminin, FN, and proteoglycans under physiologic conditions. Since FN is a main component of ECM, significant increases in FN can represent the fibrosis of ECM in many glomerulopathies including DN. TGF-*β*1 and FN increase significantly in DN and lead to glomerular sclerosis and fibrosis finally. MicroRNAs (miRNAs) are endogenously produced short noncoding RNAs of 21–25 nucleotides; they can inhibit gene expression by mRNA degradation, translation, or transcriptional inhibition [5].

miRNAs have been shown to play important roles in many cellular and biologic processes such as cell proliferation and differentiation, cancer, stress response, and development [6–8]. Recent studies have showed that miR-192 participated in the renal fibrosis in DN induced by TGF-*β*1, but the results are inconsistent. Our study aimed to investigate the relationships among miR-192, TGF-*β*1, and FN in DN with microalbuminuria and large amount of albuminuria, to explore the significance of miR-192 expression in DN.

## 2. Materials and Methods 

### 2.1. Subjects and Grouping

The subjects of experimental groups were type 2 diabetes patients admitted to the Endocrine Department of The First Hospital of China Medical University from January 2013 to October 2014. The subjects of control group were healthy persons whose age and gender were matched. Permissions were got from all the subjects before their enrollment and this paper was approved by the Ethics Institutional Review Board of China Medical University. The patients were asked about their medical history carefully including duration which indicated the time since T2DM diagnosis and their height (cm), weight (kg), and drugs.

Admission standards were as follows: type 2 diabetes patients diagnosed by OGTT according to the WHO diagnostic criteria in 1999. Patients with the following were excluded: heart diseases; hypertension; hepatic dysfunction; cancer; being pregnant; other kidney diseases (such as glomerulonephritis) or diseases of urinary system; having infection, surgery, or trauma; using agents that could affect glucose metabolism such as glucocorticoid; using agents that could affect urinary albumin excretion rate (UAER) such as ACE inhibitor or angiotensin receptor blocker.

Finally, 464 subjects were enrolled in the experimental group and they were divided into three groups according to the urine albumin creatinine ratio (UACR): normal albuminuria group (NA, UACR < 30 mg/g, *n* = 157), microalbuminuria group (MA, UACR: 30–300 mg/g, *n* = 159), and large amount of albuminuria group (LA, UACR > 300 mg/g, *n* = 148). 127 healthy persons whose age and gender were matched were selected as the control group (NC, *n* = 127).

### 2.2. Measurements of Some Parameters

All the subjects fasted at night for over 12 h. The venous blood about 5 mL of every research object was collected after 12-hour overnight fasting state and centrifuged for 15 min at 4°C with 1000 rounds/min. The supernatant was centrifuged for 5 min at 4°C with 2000 rounds/min. The supernatants were conserved at −80°C. The levels of fasting blood glucose (FBG), postprandial blood glucose (PBG), glycated hemoglobin (HbA1C), fasting insulin (FINS), postprandial insulin (PINS), fasting C peptides (FCPS), postprandial C peptides (PCPS), blood urea nitrogen (BUN), creatinine (Cr), low density lipoprotein cholesterol (LDL-C), high density lipoprotein cholesterol (HDL-C), triglyceride (TG), and cholesterol (CHO) were detected in the clinical laboratory and the endocrine experimental laboratory of The First Hospital of China Medical University.

MicroRNAs (miRNAs) were isolated from a serum volume of 400 *μ*L using the miRcute miRNA isolation kit (TianGen Biotech, Beijing, China) according to the manufacturer's instructions. Before RNA extraction,* C. elegans* synthetic miRNA cel-miR-39 miRNA Mimic (Qiagen, Hilden, Germany) was added to serum samples to correct for variations in RNA isolation derived. 5 *μ*L of miRNAs was used for reverse transcription using the miRcute miRNA First-Strand cDNA Synthesis Kit (TianGen Biotech, Beijing, China). miRcute miRNA qPCR Detection Kit (SYBR Green) (TianGen Biotech, Beijing, China) was performed using 3.0 *μ*L cDNA template under the following conditions: 94°C for 2 minutes and 45 cycles of 94°C for 5 seconds and 60°C for 40 seconds. Data analysis based on measurements of the threshold cycle was performed using the 2^−ΔΔCT^ method. ΔΔCT values are defined as the raw CT value-average of raw CT values for syn-cel-miR-39. For each sample, quantitative Real-Time PCR was performed in triplicate.

TGF-*β*1 and FN were detected by enzyme-linked immunosorbent assay (Abcam Company from China and Technoclone Company from Austria, resp.).

### 2.3. Statistical Method

The gene expression level of miR-192 was calculated with 2^−ΔΔCT^. Quantitative data that conformed to the normal distribution were reported as mean ± SDs and those that did not conform to the normal distribution were reported as median. Qualitative data were reported as percentage (%). Comparison among groups was as follows: Quantitative data that conformed to the normal distribution were compared with one-way ANOVA and those that did not conform to the normal distribution were compared with rank sum test. Qualitative data were compared with chi-square test. The correlations of any of two parameters which conformed to the normal distribution were analyzed by Pearson correlation analysis and those that did not conform to the normal distribution were analyzed by Spearman correlation analysis. The correlations of UACR and other indexes were analyzed by multiple linear regression analysis. Data were processed with the software package of SPSS 17.0. *P* < 0.05 by two-tailed test was considered to be significant.

## 3. Results 

### 3.1. Clinical Characteristics of the Subjects (Table 1)

There were significant differences in BMI, SBP, DBP, FBG, PBG, BUN, CHO, TG, LDL-C, HDL-C, and Ln (ACR) between NC group and T2DM groups (including NA, MA, and LA groups) (*P* < 0.05). Comparison among experimental groups was as follows: There were significant differences in duration, SBP, DBP, HbA1C, FBG, PBG, FINS, PINS, TG, LDL-C, HDL-C, Ln (ACR), miR-192, TGF-*β*1, and FN between NA group and MA group (*P* < 0.05). There were significant differences in duration, SBP, DBP, HbA1C, FBG, PBG, FINS, PINS, BUN, CHO, TG, LDL-C, HDL-C, Ln (ACR), miR-192, TGF-*β*1, and FN between NA group and LA group (*P* < 0.05). There were significant differences in duration, SBP, DBP, HbA1C, FCPS, BUN, CHO, TG, HDL-C, Ln (ACR), miR-192, TGF-*β*1, and FN between MA group and LA group (*P* < 0.05).

### 3.2. Comparison of miR-192, TGF-*β*1, and FN (Table 1)

There were no significant differences in miR-192, TGF-*β*1, and FN between NC group and NA group (*P* > 0.05). The expression of miR-192 in LA group was significantly lower than in MA and NA groups and the miR-192 was lower in MA group than in NA group (*P* < 0.01). The levels of TGF-*β*1 and FN in LA group were significantly higher than in MA and NA groups and those in MA group were higher in NA group than in NA group (*P* < 0.01).

### 3.3. Pearson/Spearman Correlation Analysis

In T2DM groups, the Pearson/Spearman correlation analysis showed that miR-192 (2^−ΔΔCT^) was negatively correlated with TGF-*β*1 and FN (*r* = −0.902, *P* < 0.01, and *r* = −0.797, *P* < 0.01, resp.); TGF-*β*1 was positively correlated with FN (*r* = 0.824, *P* < 0.01); Ln (ACR) was negatively correlated with miR-192 (2^−ΔΔCT^) (*r* = −0.965, *P* < 0.01), but Ln (ACR) was positively correlated with TGF-*β*1 (*r* = 0.763, *P* < 0.01), FN (*r* = 0.726, *P* < 0.01), duration (*r* = 0.502, *P* < 0.01), SBP (*r* = 0.411, *P* < 0.001), DBP (*r* = 0.302, *P* < 0.05), HbA1C (*r* = 0.465, *P* < 0.01), FBG (*r* = 0.313, *P* < 0.05), FINS (*r* = 0.362, *P* < 0.05), PCPS (*r* = 0.470, *P* < 0.01), BUN (*r* = 0.401, *P* < 0.01), Cr (*r* = 0.700, *P* < 0.01), CHO (*r* = 0.554, *P* < 0.01), TG (*r* = 0.636, *P* < 0.01), and HDL-C (*r* = −0.493, *P* < 0.01); Ln (ACR) was not correlated with age, BMI, PBG, PINS, FCPS, and LDL-C.

### 3.4. Multiple Linear Regression Analysis of Ln (UACR) and Other Parameters including miR-192, TGF-*β*1, and FN

We took Ln (UACR) as a dependent variable. Age, duration, BMI, SBP, DBP, FBG, PBG, HbA1C, FINS, PINS, FCPS, PCPS, BUN, Cr, LDL-C, HDL-C, TG, CHO, miR-192 (2^−ΔΔCT^), TGF-*β*1, and FN were selected as independent variables. The analysis showed that miR-192 (2^−ΔΔCT^), TGF-*β*1, FN, duration, and HbA1C were the independent relevant factors affecting Ln (UACR) in T2DM groups (*P* < 0.01). The regression equation is *Y* = 3.297 − 1.573*X*1 + 1.988*X*2 + 0.897*X*3 + 0.572*X*4 + 0.038*X*5 (*X*1 is miR-192 (2^−ΔΔCT^), *X*2 is TGF-*β*1, *X*3 is FN, *X*4 is duration, and *X*5 is HbA1C).

## 4. Discussion

The pathogenesis of DN is very complicated including glucose and lipid metabolism disorder, change of haemodynamics, oxidative stress, and cytokines, which can cause GBM thickening, ECM accumulation, glomerular sclerosis, filtration membrane damage, renal tubule atrophy, and renal interstitial fibrosis. These pathological changes cause urinary albumin excretion rate (UAER) increasing, slowly progressive proteinuria, and even renal dysfunction in clinical practice. Studies found recently that podocytopathy is an important cause of DN [9, 10]. Podocytes are a kind of highly differentiated cells with poor proliferation ability in glomerular basement membrane and they are an important component of glomerular filtration membrane. When the podocytes are damaged, the glomerular filtration charge barrier is weakened and albuminuria is induced. Albuminuria can increase the extracellular matrix (ECM) and accentuate renal fibrosis. Moreover, the regulation to the generation of ECM by the damaged podocytes is disordered and causes TGF-*β*1 and FN increase in DN.

TGF-*β* is a well-known cytokine that mediates the fibrosis and inflammation of kidney. TGF-*β*1 can promote the synthesis of ECM, prevent the degradation of ECM, and accumulate ECM by promoting the adhesion between cells and matrix [11]. TGF-*β*1 increase not only in the late stage, but also in the early stage of DN. Renal biopsy [4] from diabetes patients shows that the expression of TGF-*β*1 mRNA significantly increased. FN is a macromolecule glycoprotein which is the main component of ECM and it can be used to evaluate the extent of ECM accumulation [12]. FN is a main component of ECM; significant increases in FN can represent the fibrosis of ECM in many glomerulopathies including DN. Both of the synthesizing capacity of FN and the combining capacity of FN in combination with GBM increase in DM and the circulating fibronectin increased in the diabetic nephropathy [13, 14]. Basic studies found that high glucose concentration could stimulate the synthesis of FN in glomerular mesangial cells and this effect was mediated by TGF-*β*1 [15]. The FN mRNA increased in parallel with the endogenous TGF-*β*1 activity increase, and the neutralizing antibody of TGF-*β*1 could reverse this effect [16].

MicroRNAs (miRNAs) are endogenously produced short noncoding RNAs of 21–25 nucleotides. miRNAs are expressed with high tissue specificity, developmental stage specificity, and conservative property. miRNAs have been shown to play important roles in many cellular and biologic processes such as cell proliferation, differentiation and apoptosis, immune development, metabolism, virus infection, stress response, and cancer [17]. Moreover, miRNAs are expressed specifically in many diseases and different cancers such as diabetes, hepatic cancer, prostatic cancer, breast cancer, gastric cancer, squamous cell carcinoma, lymphoma, colon cancer, and lung cancer. The specific serum miRNAs phenotypes have the potential to become new kinds of diagnostic markers. miR-192 are expressed highly in kidney especially in renal cortex. Many studies have confirmed that miR-192 played important roles in the fibrosis of kidney and liver, but the conclusions are still controversial about the effect of miR-192 in DN.

Recent studies have showed that TGF-*β*1 could control the process of renal fibrosis by upregulating or downregulating several miRNAs including miR-192 [18–22]. Krupa et al. [23] found that TGF-*β*1 inhibited miR-192 expression in human proximal tubular cells (PTCs) and deficiency of miR-192 associates with renal fibrosis acceleration and GFR reduction in DN. Moreover, the expression of miR-192 was lower when the duration was longer. Wang et al. [24] also found that TGF-*β*1 decreased the expression of miR-192 in rat proximal tubular cells, mesangial cells, and human podocytes. The biopsy of diabetic patients showed that they had lower level of miR-192.

The zinc finger E-box binding homeobox-1 (Zeb1) and Zeb2 are two transcription factors located downstream of TGF-*β*1 signaling pathway which can repress E-cadherin and regulate renal fibrosis. Overexpression of miR-192 could inhibit the TGF-*β*1-mediated downregulation of E-cadherin by inhibiting the expression of Zeb1 and Zeb2 and then prevented the kidney from fibrosis. So it was reported that TGF-*β*1 inhibit the expression of miR-192, and miR-192 targeted Zeb1/2 to activate TGF-*β*1 signaling pathway and accentuated renal fibrosis in DN [23].

In our study, we found that there were no significant differences in miR-192, TGF-*β*1, and FN between NC group and NA group (*P* > 0.05). The expression of miR-192 in LA group was significantly lower than in MA and NA groups and the miR-192 was lower in MA group than in NA group (*P* < 0.01). The levels of TGF-*β*1 and FN in LA group were significantly higher than in MA and NA groups and those in MA group were higher in NA group than in NA group (*P* < 0.01). The results indicated that miR-192, TGF-*β*1, and FN could reflex the pathological progress of DN to some extent. The three parameters are significantly changed in early period of DN indicating that they may be worth for early diagnosis of DN. The multiple linear regression analysis showed that miR-192 (2^−ΔΔCT^), TGF-*β*1, and FN were the independent relevant factors affecting Ln (UACR) and also indicated that these three parameters were important factors affecting renal fibrosis process in DN. Our correlation analysis among Ln (UACR), miR-192, TGF-*β*1, and FN also showed that miR-192 was negatively correlated with TGF-*β*1, FN, and Ln (UACR); TGF-*β*1 was positively correlated with FN. These results indicated that TGF-*β*1 might downregulate miR-192, although we could not confirm the causality between them in this cross-sectional study.

However, there are several studies with opposite conclusions [25–30]. These studies find that the renal miR-192 are overexpressed in MCs and TECs of db/db mice as well as T2DM patients and miR-192 increase in parallel with increased TGF-*β*1. Deletion of inhibition of miR-192 can attenuate proteinuria and renal fibrosis and the renal function can be improved. The possible mechanisms include Smad and Akt signaling pathways. The discrepancy in these studies may be due to differences in animal species, cell types (including podocyte, mesangial cells, and renal tube cells), and experiment conditions.

## 5. Conclusions

We found that miR-192 was decreased in early DN and the levels of miR-192 were lower accompanied by the decrease of UACR. We also found that miR-192 was negatively correlated with TGF-*β*1 and FN—two parameters which represent the fibrosis extent of the kidney. These results provide us with the significance of serum miR-192 expression in clinical DN patients. The miR-192 may be the potential marker of DN diagnosis. More researches are needed to confirm the correlation of miR-192 and renal fibrosis in human DN and the underlying mechanisms.

## Figures and Tables

**Table 1:** Clinical characteristics of the subjects.

	NC	NA	MA	LA
Age (years)	51.33 ± 9.16	51.4 ± 8.57	55.40 ± 12.06	58.33 ± 9.04
Duration (years)	—	5.03 ± 1.27	8.21 ± 0.99b	11.53 ± 1.05bc
BMI (Kg/m^2^)	23.76 ± 1.60	26.49 ± 1.36a	25.30 ± 1.81a	25.31 ± 1.81a
SBP (mmHg)	115.51 ± 3.22	125.72 ± 3.43a	130.43 ± 3.04ab	136.49 ± 4.78abc
DBP (mmHg)	75.13 ± 2.64	77.67 ± 3.35a	80.21 ± 2.17ab	82.84 ± 1.73abc
HbA1C (%)	5.16 ± 0.24	8.20 ± 0.12a	8.67 ± 0.21b	8.89 ± 0.22bc
FBG (mmol/L)	5.36 ± 0.47	8.03 ± 0.60a	9.50 ± 0.34ab	9.27 ± 0.63ab
PBG (mmol/L)	6.52 (6.33–6.83)	14.46 (14.17–14.66)a	18.66 (15.4621.69)ab	16.69 (14.15–19.48)ab
FINS (mIU/L)	8.98 (8.42–9.65)	6.20 (6.15–6.25)a	7.36 (6.32–7.93)ab	7.19 (6.37–7.66)ab
PINS (mIU/L)	17.04 ± 1.04	16.59 ± 1.37	18.12 ± 1.13b	17.62 ± 1.45b
FCPS (mmol/L)	588.26 ± 4.37	590.84 ± 2.89	531.14 ± 161.08	709.38 ± 284.56c
PCPS (mmol/L)	1305.48 (1298.32–1309.87)	1388.76 (1383.28–1393.87)a	1300.00 (1295.10–1305.80)b	1513.70 (1507.60–1517.30)abc
BUN (mmol/L)	5.57 ± 1.35	6.60 ± 0.85a	6.55 ± 0.72a	7.95 ± 0.33abc
Cr (*μ*mol/L)	60.00 (54.00–72.00)	60.00 (51.00–73.00)	63.00 (57.00–70.00)	221.00 (208.00–241.00)abc
CHO (mmol/L)	4.62 ± 0.41	5.03 ± 0.40a	5.06 ± 0.33a	5.44 ± 0.44abc
TG (mmol/L)	1.39 ± 0.16	1.57 ± 0.05a	1.76 ± 0.14ab	2.11 ± 2.00abc
LDL-C (mmol/L)	2.24 ± 0.19	3.07 ± 0.22a	3.31 ± 0.21ab	3.30 ± 0.25ab
HDL-C (mmol/L)	1.20 ± 0.01	1.50 ± 0.02a	1.00 ± 0.01ab	0.80 ± 0.01abc
Ln (UACR) (mmol/L)	2.50 ± 0.06	2.87 ± 0.07a	4.88 ± 0.33ab	7.11 ± 0.91abc
miR-192 (2^−ΔΔCT^) (ng/mg)	1.00 ± 0.02	0.99 ± 0.01	0.63 ± 0.01ab	0.34 ± 0.01abc
TGF-*β*1 (ng/mL)	6.49 ± 0.24	11.61 ± 0.32e	20.35 ± 0.97ef	28.95 ± 1.28efg
FN (*μ*g/mL)	85.67 ± 2.08	99.71 ± 2.65e	166.8 ± 13.42ef	245.83 ± 9.04efg

a: compared with NC group, *P* < 0.05; b: compared with NA group, *P* < 0.05; c: compared with MA group, *P* < 0.05; e: compared with NC group, *P* < 0.01; f: compared with MA group, *P* < 0.01; g: compared with MA group, *P* < 0.01. “—” presents no data.

BMI: body mass index; SBP: systolic blood pressure; DBP: diastolic blood pressure; HbA1C: glycated hemoglobin; FBG: fasting blood glucose; PBG: postprandial blood glucose; FINS: fasting insulin; PINS: postprandial insulin; FCPS: fasting C peptides; PCPS: postprandial C peptides; BUN: blood urea nitrogen; Cr: creatinine; CHO: cholesterol; TG: triglyceride; LDL-C: low density lipoprotein cholesterol; HDL-C: high density lipoprotein cholesterol; Ln (UACR): logarithm of urine albumin creatinine ratio; TGF-*β*1: transforming growth factor; FN: fibronectin.
